# Association between health information, use of protective devices and occurrence of acute health problems in the Prestige oil spill clean-up in Asturias and Cantabria (Spain): a cross-sectional study

**DOI:** 10.1186/1471-2458-6-1

**Published:** 2006-01-03

**Authors:** José Miguel Carrasco, Virginia Lope, Beatriz Pérez-Gómez, Nuria Aragonés, Berta Suárez, Gonzalo López-Abente, Fernando Rodríguez-Artalejo, Marina Pollán

**Affiliations:** 1 Environmental Epidemiology and Cancer Unit, National Centre for Epidemiology, Carlos III Institute of Health, Madrid, Spain; 2 Department of Preventive Medicine and Public Health, School of Medicine, Autonomous University of Madrid, Madrid, Spain

## Abstract

**Background:**

This paper examines the association between use of protective devices, frequency of acute health problems and health-protection information received by participants engaged in the Prestige oil spill clean-up in Asturias and Cantabria, Spain.

**Methods:**

We studied 133 seamen, 135 bird cleaners, 266 volunteers and 265 paid workers selected by random sampling, stratified by type of worker and number of working days. Information was collected by telephone interview conducted in June 2003. The association of interest was summarized, using odds ratios (OR) obtained from logistic regression.

**Results:**

Health-protection briefing was associated with use of protective devices and clothing. Uninformed subjects registered a significant excess risk of itchy eyes (OR:2.89; 95%CI:1.21–6.90), nausea/vomiting/dizziness (OR:2.25; 95%CI:1.17–4.32) and throat and respiratory problems (OR:2.30; 95%CI:1.15–4.61). There was a noteworthy significant excess risk of headaches (OR:3.86: 95%CI:1.74–8.54) and respiratory problems (OR:2.43; 95%CI:1.02–5.79) among uninformed paid workers. Seamen, the group most exposed to the fuel-oil, were the worst informed and registered the highest frequency of toxicological problems.

**Conclusion:**

Proper health-protection briefing was associated with greater use of protective devices and lower frequency of health problems. Among seamen, however, the results indicate poorer dissemination of information and the need of specific guidelines for removing fuel-oil at sea.

## Background

On 13 November 2002, the single-hulled petrol tanker, Prestige, carrying 77,033 tonnes of heavy fuel, suffered serious damage requiring the evacuation of the crew. Six days later, the ship broke in two off the south-west coast of Finisterre, Galicia (Spain), and sank to a depth of 3,500 metres (approximately 11,500 ft.) [1]. The accident led to a major spill of the tanker's cargo of oil, with the first black oil-laden tide arriving on the Galician coast on 16 November. By early December, the oil had spread, and started coming ashore on the Asturian coast and, subsequently, in Cantabria and the Basque Country, thereby affecting the entire northern coast of Spain [2].

The heavy fuel (listed as M100, No. 6 or No. 2 according to the Russian, Anglo-Saxon and French classifications respectively) [3,4] discharged by the Prestige contains three groups of substances potentially hazardous to health, i.e., volatile organic compounds (VOCs), polycyclic aromatic hydrocarbons (PAHs) and heavy metals, particularly zinc, nickel and vanadium. Furthermore, it has a density of 992.1 kg/m3 at 15°C (11.04° API), a viscosity of 615 centiStockes at 50°C and a low tendency to evaporate and disperse naturally [3].

The Ministry of Health & Consumer Affairs, acting in liaison with the Asturian and Cantabrian Public Health Authorities, sponsored a survey intended: firstly, to characterise exposure to fuel-oil by persons participating in the oil spill clean-up; secondly, to study the use of protective devices and health-protection information received; and, thirdly, to ascertain the acute health problems experienced by such participants. As a result of this project, acute health problems experienced by the persons who co-operated in the clean-up tasks, and the association between such problems and the nature of the work and use of protective devices in the regions of Asturias and Cantabria [5] were analysed. These individuals were basically divided into four groups according to the type of work undertaken, i.e., volunteers, bird cleaners, seamen and purpose-paid workers [5]. Briefly, the volunteers generally worked on weekends, in both high- and low-pollution areas, devoting themselves almost exclusively to cleaning up of boulders and rocks, shingle beaches, sandy beaches and wharves. The bird cleaners performed their tasks, also generally for short periods (weekends), in closed premises where they received the oil-coated birds. The seamen worked in highly polluted areas, positioning floating barriers and booms, and skimming up the oil from their boats, for periods that generally exceeded 20 days [5]. Finally, the purpose-paid workers patrolled highly polluted areas of coastline, carrying out boulders and rocks, shingle beaches, sandy beaches, wharves and high-pressure/vacuum clean-up activities. Like the seamen, their work periods were longer than those of volunteers and bird cleaners. Although there was a high percentage of use and few tears and breakages of protective equipment in all groups, special mention must nevertheless be made of the high proportions of torn gloves among bird cleaners and of torn suits among seamen, in particular, who, moreover, reported wearing masks to a much lesser extent than the other groups [5].

An important result of this first study was the greater frequency of disorders among seamen, and the negligible magnitude of the difference between paid workers and volunteers vis-à-vis the frequency of health problems, despite the fact that paid workers and seamen were involved for an average of two months, whereas volunteers participated for less than a week. These data might suggest that the frequency of health problems could be associated with differences in the health-protection information received. Health-protection information can be an important resource in risk prevention in the case of clean-up workers and, in some contexts, may be less costly than other preventive measures. Nevertheless, the usefulness of a message cannot be taken for granted: not only must it be communicated in an understandable and trustworthy manner, but it should also capture the attention of and be perceived as useful, effective, and acceptable by the target audience [6-9].

Owing to the possible health risk associated with the Prestige oil spill clean-up work, those involved in such tasks received health-protection information. In Asturias and in Cantabria, information was disseminated by a number of public administrative bodies (regional authorities, as well as city and town councils), Civil Protection Corps, fishermen's guilds, some non-governmental organisations (Red Cross, ecologist associations) and the companies (*TRAGSA*; *Empresa de Residuos de Cantabria*) contracted by the Regional Authorities to clean up the beaches and remove the oil residue and tar. In general, the information furnished was based on the *Regulations for the Prevention of Risk in the Cleaning up of Areas Polluted by the Oil Spill from the vessel "Prestige" *(*Normas para la prevención de los riesgos en las tareas de limpieza de zonas contaminadas por el vertido de Fuel-Oil del Buque "Prestige"*) issued by the Ministry of Health & Consumer Affairs (*Ministerio de Sanidad y Consumo*-regulations available on request-). These regulations include individual protection measures (work clothes, protective goggles, gloves, boots and mask), recommendations as to diet and hygiene, and a series of circumstances that contraindicate the work for certain persons. Briefings were mainly oral and, as the groups were to be allocated different tasks, each tended to receive specific information, different to that given to the others.

Paid workers were the group that received the most uniform briefings. Most workers were briefed by the TRAGSA Risk Prevention Unit, which issued a series of regulations, containing general information on occupational risk prevention, as well as specific information on removing residue from beaches, cleaning rocky stretches of coastline with high-pressure jets and hoses, conducting spill surveillance of slicks approaching beaches, and using self-propelled sand-rake and beach-cleaning equipment. This information was explained by a prevention technician and a talk was given to each work party prior to the activity. Some workers were hired directly by the town councils affected, and in such cases it was the council itself that undertook the necessary briefing.

Bird cleaners received the Ministry guidelines plus specific recommendations as regards the working conditions at the San Juan de Nieva Bird Rescue & Recovery Centre (e.g., direct work with animals at high temperatures). This information was mainly supplied by Asturias Health Authority staff and ecological associations.

Among seamen and volunteers the information received was more heterogeneous. The seamen were mainly briefed by fishermen's guilds, which the Cantabrian Regional Authority had supplied with a set of "Measures to be adopted by persons engaged in hydrocarbon clean-up work at sea". These measures include: circumstances that contraindicate the work for certain persons; guidelines regarding the use of individual protective equipment (goggles, dungarees, mask, gloves, boots and protective suit); recommendations in the event of occasional direct contact with fuel-oil; recommendations on the consumption of food and drink; cleanliness of equipment; description of symptoms and effects due to prolonged exposure; and first aid. Volunteers were informed by a series of different institutions, with a high degree of participation by NGOs. The information furnished was mainly drawn from the above-mentioned ministerial guidelines.

In the above context, this paper sought to examine the association between use of protective devices, frequency of acute health problems and receipt of the pertinent health-protection information prior to performing clean-up tasks following the Prestige oil spill among above mentioned four groups of people engaged in clean-up activities in Asturias and Cantabria, namely volunteers, paid workers, seamen and bird cleaners.

## Methods

Selection of the study sample and data-collection have both been described in an earlier paper [5]. The study population comprised persons who participated in the clean-up of the pollution caused by the Prestige and were registered in the censuses taken by Public Health Authorities of Asturias and Cantabria. This census information included full name, date of birth, group, number of days worked and telephone number. After excluding persons with no information on number of days worked and those who had formed part of 2 or more groups, the sampling framework was made up of 4117 persons in Asturias and 3621 in Cantabria. No seamen were included in the Asturian census and only two bird cleaners (who were not interviewed) were registered in the Cantabrian census.

The health authorities decided *a priori *to include a total of 400 persons in each of the two geographic areas, viz., Asturias and Cantabria. Initially, 100 persons were to be included in each group and area, but, given the absence of seamen in the Asturian worker census and the lack of bird cleaners in Cantabria, it was decided that the sample size of each group would be increased to 133 in order to maintain the total sample at 400 workers per geographic area. Samples were separately selected for Asturias and Cantabria by means of random sampling stratified by two variables, i.e., "group affiliation" (volunteers, paid workers, seamen and bird cleaners) and "number of cleaning days worked as a member of that group" (less or more than five days), in order to favour the overrepresentation of individuals who had cleaned for longer periods. The final study sampling comprised 133 seamen in Cantabria, 135 bird cleaners in Asturias, and 266 volunteers and 265 paid workers in both regions together. The corresponding number of subjects was predetermined by stratum, and a main and two substitute samples were extracted from the census, randomly establishing a one-to-one relationship between units of the main and each of the substitute samples to reduce any bias caused by replacements. A total of 62.5% of persons selected and located in the main sample agreed to participate in the study. Individuals who could not be contacted after three attempts on different days and at different times of day, or who did not wish to participate were replaced by the relevant substitute. As the composition of the sample was not proportional to the study population, all estimates were computed using the corresponding weighting factors (StataCorp., 2004).

Data for the epidemiological survey conducted by the Ministry of Health & Consumer Affairs were collected by telephone interview during the first 20 days of June 2003. The structured questionnaire was based on one that had been previously used in France after the Erika oil spill [10] and included data on type and duration of clean-up activity, use of protective devices, contact with oil-fouled products, perceived health problems, alternative exposures to PAHs and health-protection information received. The data obtained on items relating to health-protection information were used for this study.

For study purposes, an informed person was defined as any subject who had received information before the start of the clean-up activity. Health problems were divided into two major groups, namely, injuries and toxicological problems. The former grouped together the consequences of physical work, e.g., low back pain and lesions (bruises, scratches, blisters, superficial or deep cuts, twists and sprains, broken bones, knee pain and chipped teeth). Toxicological effects included symptoms previously related to exposure to VOCs and PAHs, such as headaches, itchy eyes, throat and respiratory tract problems and nausea/vomiting/dizziness symptoms (including any of them). Differences in proportion were analysed using the Chi-squared test. The association between reported health problems and information received was summarized using odds ratios (OR) and their 95% confidence intervals, obtained from logistic regression. Odds ratios adjusted for time worked in high- and low-pollution areas were likewise obtained. Analyses were performed independently for each group because time of exposure, tasks performed and data sources varied accordingly.

## Results

The characteristics of the health-protection information received by the different groups of clean-up workers are shown in Table 1. Most workers reported having received information, with paid workers accounting for the highest and seamen for the lowest percentages (94% and 68% respectively). Essentially, this information was imparted orally, prior to beginning the activity. In the case of paid workers, the waste-removal company was the most usual source of information (58%). Volunteers were mainly informed by Regional Health Authority staff (31%) and other sources (37%), principally the Civil Protection Corps (*Protección Civil*) and fire brigade. In the case of seamen, information was furnished in most cases by the fishermen's guilds, whilst Health Authority staff (35%), in tandem with other volunteers and ecologist organisations (32%), focused on briefing the bird cleaners. The information received was deemed useful by the great majority of subjects, with bird cleaners accounting for the highest percentage (97%).

Table 2 shows the frequency of acute health problems reported. Seamen were the group with the highest prevalence of symptoms, mostly in the form of headaches (28%) and throat and respiratory tract problems (30%). While headaches (16%) and nausea/vomiting/dizziness (15%) tended to be frequent among paid workers, nausea/vomiting/dizziness (10%) and lesions (19%) were the main cause for complaint among volunteers and bird cleaners respectively.

Table 3 shows the percentage of use and breakage/tear of protective devices among informed and uninformed subjects. In comparison with uninformed paid workers, those who received health-protection information reported greater use of safety goggles (88% versus 70%) and fewer broken boots (0% versus 4%). In the volunteer group, informed subjects reported having worn the protective suit more frequently than their uninformed counterparts (85% versus 66%), and having experienced fewer torn protective suits (18% versus 45%) and broken masks (1% versus 9%). Differences in use and breakage/tear of protective devices were not significant among bird cleaners and seamen. However, attention should be drawn to the greater use of safety goggles and masks among informed seamen, the high proportion of tears to protective suits among seamen in general, and the scant use of protective clothing among bird cleaners.

Table 4 shows the association between health-protection briefing and prevalence of self-reported acute health problems. The results, adjusted for days worked in high- and low-pollution areas, show that uninformed subjects registered an excess risk for all reported symptoms, which proved statistically significant in the case of itchy eyes (OR:2.67; 95%CI:1.13–6.28), nausea/vomiting/dizziness (OR:2.09; 95%CI:1.07–4.08), and throat and respiratory problems (OR:2.08; 95%CI:1.02–4.24). Uninformed paid workers registered a statistically significant increased risk of lower back pain (OR:4.28; 95%CI:1.53–12.02), headaches (OR:3.58; 95%CI:1.55–8.24) and an excess -close on statistically significant- risk of throat and respiratory tract problems (OR:2.29; 95%CI:0.95–5.54). Among uninformed volunteers, there was an excess -close on statistically significant- risk of throat and respiratory tract problems (OR:3.17; 95%CI:0.96–10.50). Uninformed bird cleaners displayed a high risk of lower back pain (OR:9.29; 95%CI:1.14–75.55) and itchy eyes (OR:18.37; 95%CI:2.58–130.76), though this was based on only 3 and 4 observed uninformed cases. Lastly, differences in health problems reported by informed and uninformed seamen were not significant.

## Discussion

To our knowledge, this is the first study to analyse the importance of health information supplied to workers involved in clean-up operations following a massive oil spill. This study shows that most participants in the Prestige oil spill clean-up received health-protection information, mainly in the form of an oral briefing given prior to the start of activity. In general, subjects who were informed reported a higher frequency of use and a lower percentage of broken/torn protective devices, along with a lower frequency of acute health problems than did subjects who were not informed. This pattern of behaviour may indicate successful communication of health-risk information [7].

There is abundant literature on communicating health risk information guidelines [6-9]. However, manuscripts assessing the effect of preventive information in specific settings such as ours are relatively scarce; effectiveness of preventive health information has been studied, for example, in environmental health disasters [11], in epidemiological outbreaks [12] or in occupational settings[13], showing the importance of developing strategies orientated to diminish the risks.

Some methodological aspects of our study call for comment. Firstly, the fact that health-protection information was disseminated before the commencement of clean-up activity means that we were able to establish a temporal relationship between health briefing on the one hand, and use of protective devices and occurrence of acute health problems, on the other. Secondly, self-report is the appropriate procedure for collecting data on the occurrence of symptoms in cases where objective diagnosis is not possible. Furthermore, as most workers' injuries did not require health care, diagnoses could not be verified against medical information. Thirdly, the telephone interview is a simple and valid system for collecting data in this context. Indeed, a number of studies have reported that, in the case of behavioural risk factors and implementation of preventive practices, telephone interviews yield results comparable to those of face-to-face [14,15] or self-administered [16] surveys.

This study also has certain limitations, which have to be borne in mind to ensure correct interpretation of the results. The possible existence of some degree of observation bias cannot be ruled out, since the public outcry linked to the spill and the ensuing financial loss might well have influenced participants' replies. It should be noted, however, that there was far less alarm in the geographic area targeted by this study, because other parts of the country (Galicia) had been more severely affected previously and the local authorities were consequently that much better prepared. Moreover, the interviews were conducted six months after the arrival of the oil and the economically affected parties were subsequently compensated. A further aspect to be taken into account is the possibility that some of the reported associations might be the consequence of chance, given the number of statistical tests performed in the course of analysing the data. Nevertheless, attention must be drawn to the high consistency of the associations that emerge from the tables, something that supports the results obtained. Moreover, the limited sample size of the uninformed group endows our study with a low statistical power. Notwithstanding this, significant briefing-related differences were detected, both in the use of protective devices and in the frequency of health problems, specifically those linked to toxic components contained in the fuel-oil.

Our results show that health-protection information was provided to most workers. As regards the channel of communication, the fact that subjects were briefed orally probably means: that there was greater access to and better comprehension of the information; and that, overall, this might have contributed to the recommendations and regulations imparted being viewed as beneficial by over 84% of interviewees. A higher proportion of paid workers enjoyed access to such information, probably because they were employed by a waste removal company, with legal obligations relating to occupational safety and hygiene, and more structured protocols for prevention of occupational injuries and warnings about risks. In contrast, seamen reported a notably lower percentage of informed subjects than did the other three groups of clean-up workers.

With regard to the use of protective devices, our results indicate that, in general, informed subjects used such devices -safety goggles in particular- more than did their uninformed fellow workers, and that they experienced fewer tears and breakages. Although the information on the use of protective devices was probably clear, it would nonetheless appear to have been more effective among paid workers and volunteers. Unlike other studies undertaken in similar spills [10,17], the low frequency of skin irritation observed in this study might be explained by the proper and frequent use of the protective devices and clothing supplied.

The data attest to the benefit of furnishing information on the prevention of acute health problems -particularly those of a toxicological nature- in the performance of this type of task. This could be due to the effectiveness of protective devices as a barrier against exposure, while the risk of suffering injury must be assumed to be determined, to a certain degree, by the skill of the individual subject.

Seamen were essentially involved in clean-up tasks at sea, where VOC and PAH concentrations are highest. Earlier studies have shown that direct contact with these products can cause acute health problems, such as neurological disorders (headaches, nausea, dizziness and somnolence) in the case of exposure to VOCs, and respiratory difficulty, digestive problems (nausea, vomiting and abdominal pain) and itchy eyes and skin in the case of PAHs [18]. Indeed, this was the group that reported the most health problems, the least use of masks, and a higher frequency of tears to protective suits. Furthermore, almost half of the seamen reported having eaten while in contact with fuel-oil [5]. Yet it is relevant to point out that there were no significant differences in the frequency of health problems among informed and uninformed seamen. These results indicate that the information campaign should have been on a much larger scale among seamen and highlight the need for specific protection measures for this group, which performed its clean-up tasks in a setting that was different and entailed a higher probability of exposure. Special mention must also be made of the high percentage of lesions reported by bird cleaners owing, presumably, to the highly specific nature of the tasks performed. The possibility of preventing such injuries depends, above all, on the skill of the person responsible, since gloves are powerless to prevent many of these injuries.

## Conclusion

In conclusion, the information received by workers engaged in the clean-up of the Prestige oil spill in Asturias and Cantabria was associated with a greater use of individual protective devices and lower frequency of acute health problems, mainly among the volunteers and paid workers. The experience gained and the health problems detected along the Galician coast may well have served to guide the protection and prevention actions applied in the clean-up operations in Asturias and Cantabria, regions that were affected at a later point in time. It should be stressed, however, that it was seamen, who were the poorest informed, suffered the most toxicological problems (perhaps as a consequence of the scant use of masks) and constituted the subset among whom the information received was least effective. Hence, were a similar situation to arise, this group should arguably receive attention specifically tailored to its designated activities and the conditions under which it works.

## Competing interests

The author(s) declare that they have no competing interests.

## Authors' contributions

JMC and VL conceived the idea, carried out the statistical analysis and wrote the manuscript. BPG, NA, BS and GLA made contribution to statistical analyses and interpretation of results, and revised the manuscript for important intellectual content. FRA and MP designed the study, contributed to manuscript writing, and revised it for important intellectual content. All authors contributed to the final version of the manuscript.

## Pre-publication history

The pre-publication history for this paper can be accessed here:



## Figures and Tables

**Table 1:** Characteristics of the health-protection information received by workers engaged in the clean-up of the Prestige oil-spill. Number of subjects in the sample and estimated percentage in the population.

	**Paid workers** **(N = 265)**		**Volunteers** **(N = 266)**		**Seamen** **(N = 133)**		**Bird cleaners** **(N = 135)**		**p**
	**n**	**%**	**n**	**%**	**n**	**%**	**n**	**%**	
					
**Information received**									
No	15	5.9	33	12.9	43	32.2	17	12.8	
Yes	250	94.1	233	87.2	90	67.9	118	87.2	<0.001
**Time**									
Prior to the activity	221	88.9	229	98.4	84	93.3	114	96.2	
During the activity	29	11.1	4	1.6	6	6.7	4	3.8	<0.001
**Manner**									
Written	48	18.9	53	22.2	20	22.2	3	2.5	
Orally	202	81.1	180	77.8	70	77.8	115	97.5	<0.001
**Briefing given by**									
Public Health Authority staff	16	7.9	55	31.2	14	20.2	38	35.3	
City or town council staff	40	18.0	35	19.0	1	1.4	13	12.4	
Waste removal company staff	112	57.7	6	3.5	11	14.9	6	4.8	
Volunteers, ecologist organisations	2	0.9	17	9.0	1	1.4	40	32.0	
Others^1^	32	15.5	67	37.4	43	62.0^2^	17	14.7	<0.001
**Perceived utility**									
Not useful	25	9.7	25	9.6	14	15.6	4	3.0	
Useful	225	90.3	208	90.4	76	84.4	114	97.0	0.028

^1^Civil Protection Corps, fire brigade, 112 (Spanish general emergency telephone)-service personnel, Red Cross, Dept. of the Environment and fishermen's guilds.

^2^Mostly briefed by the fishermen's guilds.

**Table 2:** Acute health problems reported by workers engaged in the clean-up of the Prestige oil-spill. Number of subjects in the sample and estimated percentage in the population.

	**Paid workers** **(N = 265)**		**Volunteers** **(N = 266)**		**Seamen** **(N = 133)**		**Bird cleaners** **(N = 135)**		**p**
	**n**	**%**	**n**	**%**	**n**	**%**	**n**	**%**	
					
**Injuries**									
Lesions	23	8.7	9	3.2	10	7.6	31	19.2	<0.001
Lower back pain	17	6.1	16	5.6	12	9.1	5	3.1	0.281
**Toxicological symptoms**									
Headaches	40	15.8	17	5.7	38	28.4	8	5.0	<0.001
Itchy eyes	17	7.1	11	4.6	12	9.1	6	4.0	0.295
Nausea/vomiting/dizziness	38	15.4	24	9.5	21	15.4	16	11.2	0.169
Throat and respiratory tract problems	30	12.4	18	5.6	40	30.4	12	8.0	<0.001

**Table 3:** Estimated percentage of use and breakage of protective devices according to health-protection information received.

	**Total** **(N = 799)**			**Paid workers** **(N = 265)**			**Volunteers** **(N = 266)**			**Seamen** **(N = 133)**			**Bird cleaners** **(N = 135)**		
					
	**Inform.^1 ^** **(N = 648)**	**Uninf.^2 ^** **(N = 151)**		**Inform.^1^** **(N = 221)**	**Uninf.^2 ^** **(N = 44)**		**Inform.^1^** **(N = 229)**	**Uninf.^2 ^** **(N = 37)**		**Inform.^1^** **(N = 84)**	**Uninf.^2 ^** **(N = 49)**		**Inform.^1^** **(N = 114)**	**Uninf.^2 ^** **(N = 21)**	
	**%**	**%**	**p**	**%**	**%**	**p**	**%**	**%**	**p**	**%**	**%**	**p**	**%**	**%**	**p**
					
**Suit**															
Used	77.5	64.8	0.017	87.6	87.8	0.973	84.7	65.6	0.008	88.8	85.5	0.580	35.6	28.9	0.559
Torn	33.1	59.5	<0.001	36.3	44.5	0.351	18.2	45.4	0.003	75.7	81.0	0.512	8.9	28.4	0.165
**Gloves**															
Used	99.4	98.4	0.126	98.5	97.5	0.666	100.0	100.0	-	93.6	95.9	0.588	99.0	94.8	0.194
Torn	13.2	17.0	0.228	11.8	11.8	0.987	7.2	16.7	0.061	6.4	8.5	0.660	31.8	44.6	0.279
**Safety goggles**															
Used	76.2	63.2	0.014	88.0	70.3	0.006	79.6	65.2	0.062	72.0	56.5	0.073	54.0	55.6	0.894
Broken	0.6	1.1	0.622	1.0	2.6	0.398	0.0	0.0	-	0.0	0.0	-	1.1	0.0	0.662
**Protective hat**															
Used	15.5	11.2	0.300	13.8	18.8	0.452	17.2	11.4	0.356	12.0	4.1	0.135	10.0	10.3	0.959
Torn	0.0	0.0	-	0.0	0.0	-	0.0	0.0	-	0.0	0.0	-	0.0	0.0	-
**Mask**															
Used	91.7	90.0	0.567	95.1	87.8	0.071	91.0	93.5	0.613	74.4	62.8	0.161	96.8	100.0	0.393
Broken	1.0	3.2	0.057	1.1	2.1	0.588	0.7	9.4	<0.001	0.0	0.0	-	1.0	0.0	0.654
**Boots**															
Used	88.7	91.2	0.410	98.0	93.9	0.116	99.0	100.0	0.559	93.6	95.9	0.588	36.1	52.6	0.166
Broken	0.4	1.5	0.123	0.4	3.9	0.016	0.0	0.0	-	1.3	0.0	0.436	0.0	0.0	-

^1^Percentage of informed persons at the time of commencement of the activity.

^2^Percentage of uninformed persons or informed during the activity.

**Table 4:** Association of health-protection information received with frequency of acute health problems.

	**Total (N = 799)**					
	
**Symptoms**	Uninformed^1 ^ (N = 151)	Informed (N = 648)	Univariate analysis^2^		Multivariate analysis^2^	
	Cases	Cases	OR^3^	95%CI^3^	OR^4^	95%CI^4^
	
Lesions	15	58	1.67	0.75 – 3.72	1.53	0.68 – 3.41
Lower back pain	18	32	1.38	0.61 – 3.12	1.32	0.56 – 3.10
Headaches	33	70	1.77	0.90 – 3.50	1.66	0.83 – 3.34
Itchy eyes	18	28	2.89	1.21 – 6.90	2.67	1.13 – 6.28
Nausea/vomiting/dizziness	30	69	2.25	1.17 – 4.32	2.09	1.07 – 4.08
Throat and respiratory problems	32	68	2.30	1.15 – 4.61	2.08	1.02 – 4.24
						
	**Paid workers (N = 265)**					
	
	Uninformed^1 ^ (N = 44)	Informed (N = 221)	Univariate analysis^2^		Multivariate analysis^2^	
	Cases	Cases	OR^3^	95%CI^3^	OR^4^	95%CI^4^
	
Lesions	2	21	0.43	0.09 – 1.97	0.39	0.08 – 1.92
Lower back pain	8	9	5.04	1.78 – 14.28	4.28	1.53 – 12.02
Headaches	15	25	3.86	1.74 – 8.54	3.58	1.55 – 8.24
Itchy eyes	4	13	1.28	0.38 – 4.29	1.28	0.39 – 4.16
Nausea/vomiting/dizziness	11	27	2.18	0.92 – 5.20	2.26	0.97 – 5.22
Throat and respiratory problems	10	20	2.43	1.02 – 5.79	2.29	0.95 – 5.54
						
	**Volunteers (N = 266)**					
	
	Uninformed^1 ^ (N = 37)	Informed (N = 229)	Univariate analysis^2^		Multivariate analysis^2^	
	Cases	Cases	OR^3^	95%CI^3^	OR^4^	95%CI^4^
	
Lesions	3	6	2.58	0.56 – 11.97	2.57	0.56 – 11.80
Lower back pain	1	15	0.50	0.06 – 3.95	0.57	0.07 – 5.01
Headaches	4	13	1.48	0.39 – 5.61	1.70	0.44 – 6.65
Itchy eyes	3	8	2.53	0.63 – 10.15	2.24	0.55 – 9.05
Nausea/vomiting/dizziness	7	17	2.49	0.92 – 6.73	2.23	0.79 – 6.28
Throat and respiratory problems	5	13	2.96	0.91 – 9.64	3.17	0.96 – 10.50
						
	**Seamen (N = 133)**					
	
	Uninformed^1 ^ (N = 49)	Informed (N = 84)	Univariate analysis^2^		Multivariate analysis^2^	
	Cases	Cases	OR^3^	95%CI^3^	OR^4^	95%CI^4^
	
Lesions	5	5	1.81	0.49 – 6.71	1.91	0.52 – 7.08
Lower back pain	6	6	1.83	0.55 – 6.11	2.16	0.66 – 7.07
Headaches	11	27	0.64	0.28 – 1.46	0.58	0.25 – 1.35
Itchy eyes	7	5	2.65	0.78 – 9.02	2.75	0.78 – 9.74
Nausea/vomiting/dizziness	7	14	0.89	0.33 – 2.43	0.82	0.29 – 2.30
Throat and respiratory problems	13	27	0.77	0.35 – 1.70	0.69	0.31 – 1.54
						
	**Bird cleaners (N = 135)**					
	
	Uninformed^1 ^ (N = 21)	Informed (N = 114)	Univariate analysis^2^		Multivariate analysis^2^	
	Cases	Cases	OR^3^	95%CI^3^	OR^4^	95%CI^4^
	
Lesions	5	26	1.59	0.54 – 4.74	1.30	0.37 – 4.58
Lower back pain	3	2	7.89	1.13 – 55.33	9.29	1.14 – 75.55
Headaches	3	5	4.47	0.93 – 21.55	4.51	0.83 – 24.46
Itchy eyes	4	2	12.23	1.91 – 78.42	18.37	2.58 – 130.76
Nausea/vomiting/dizziness	5	11	2.73	0.80 – 9.35	2.52	0.70 – 9.06
Throat and respiratory problems	4	8	3.62	0.94 – 14.01	3.65	0.69 – 19.35

^1 ^Includes subjects informed after the activity had commenced.

^2 ^Estimators extracted from logistic regression including the corresponding symptom as the dependent variable, and briefing status as the explanatory variable.

^3 ^OR – (95%) CI = odds ratio (uninformed versus informed) – 95% confidence interval.

^4 ^OR – (95%) CI = odds ratio (uninformed versus informed) adjusted for time worked in high- and low-pollution areas -95% confidence interval.
